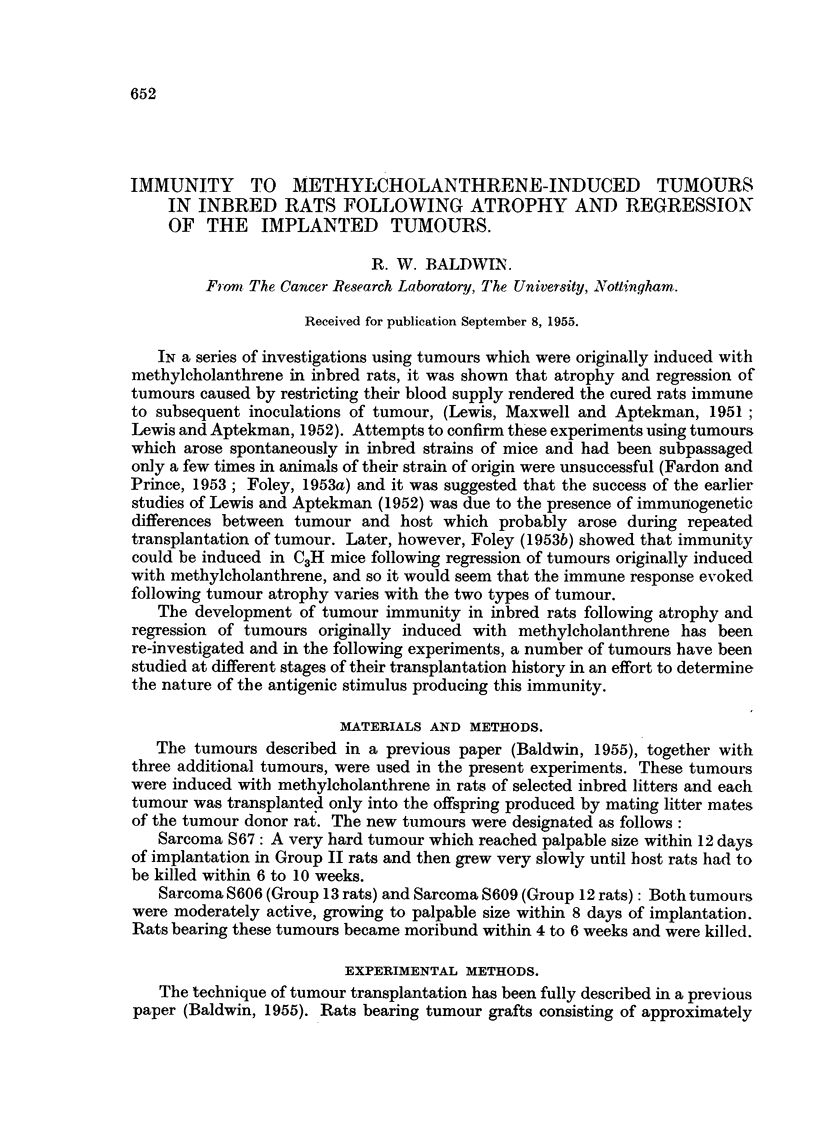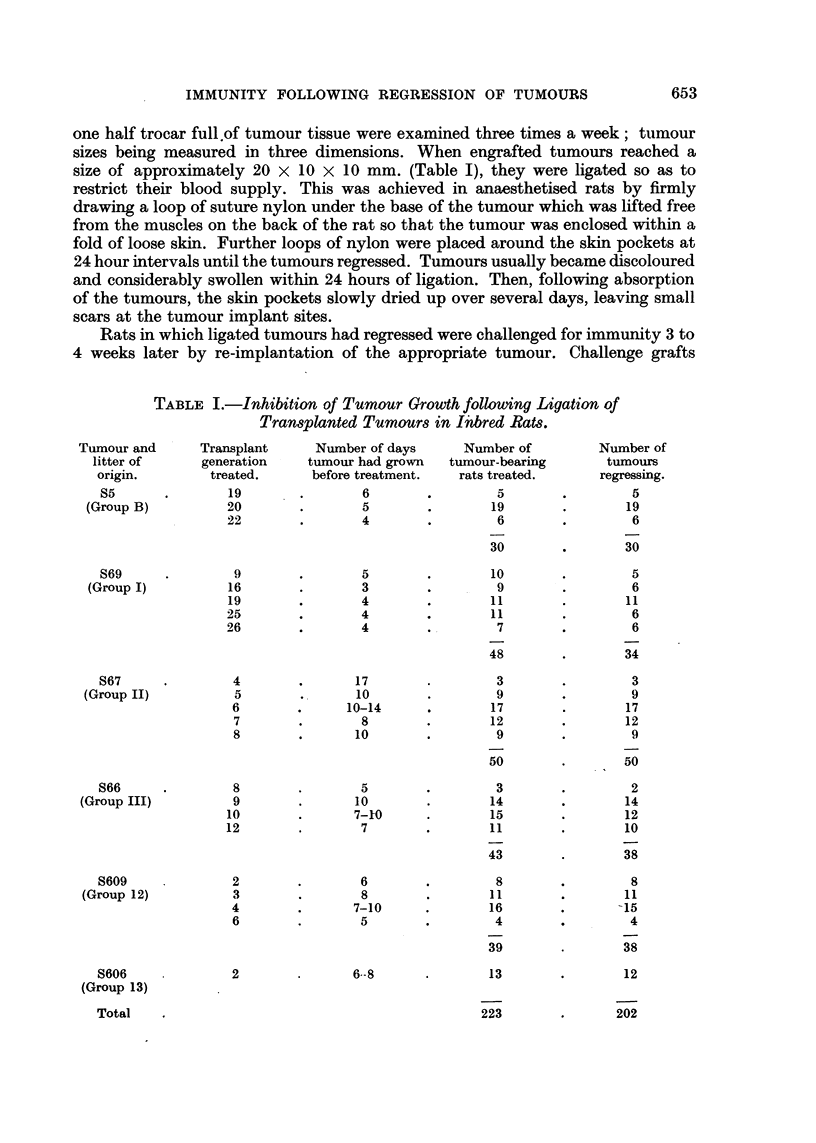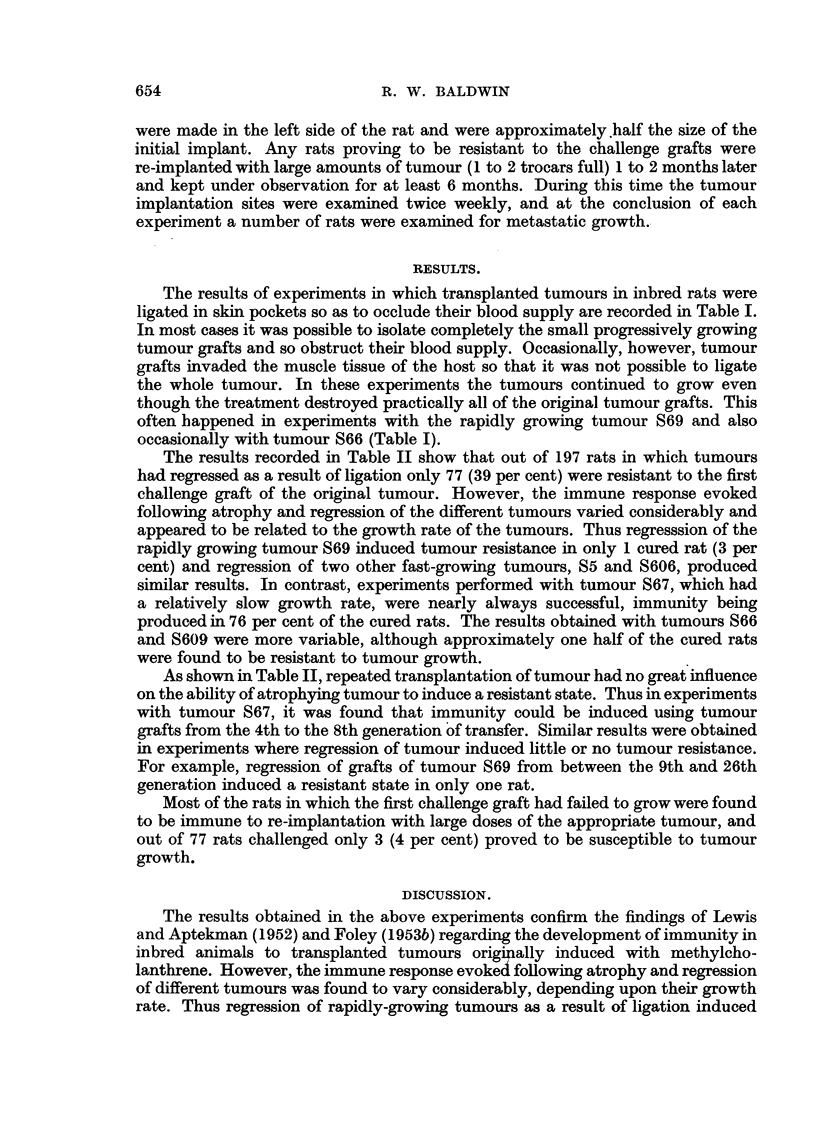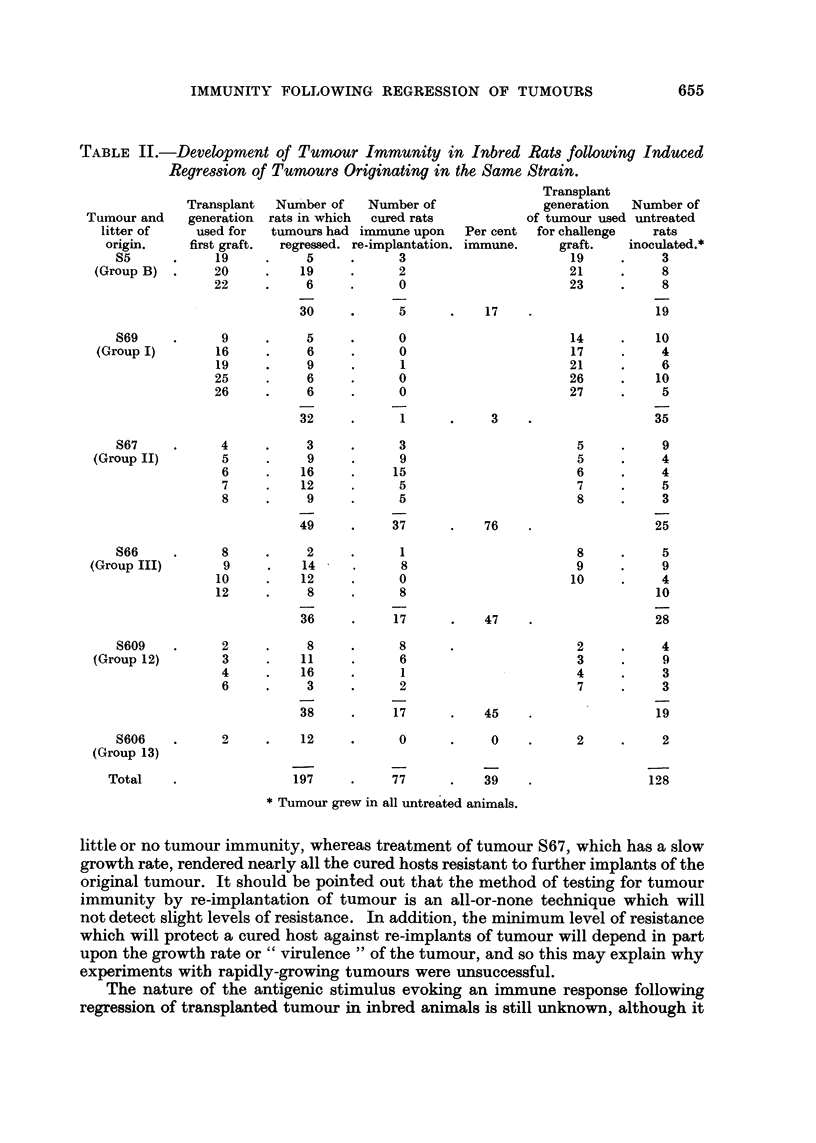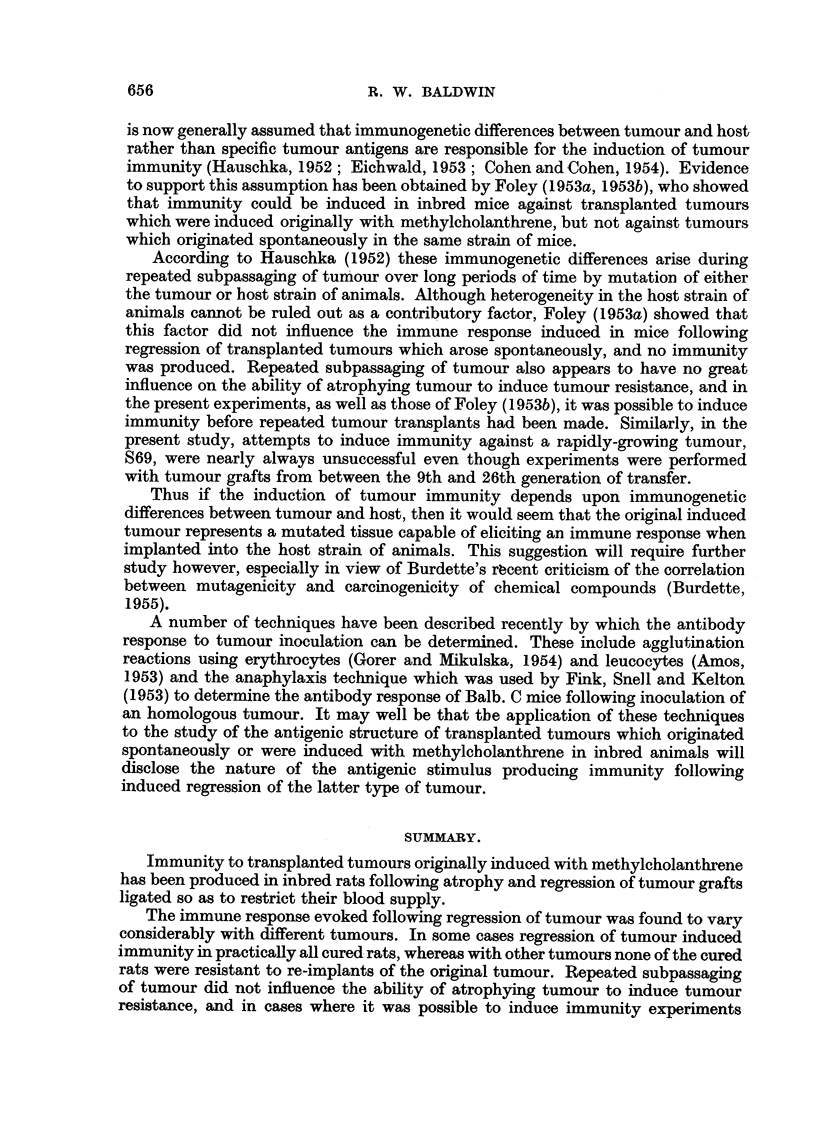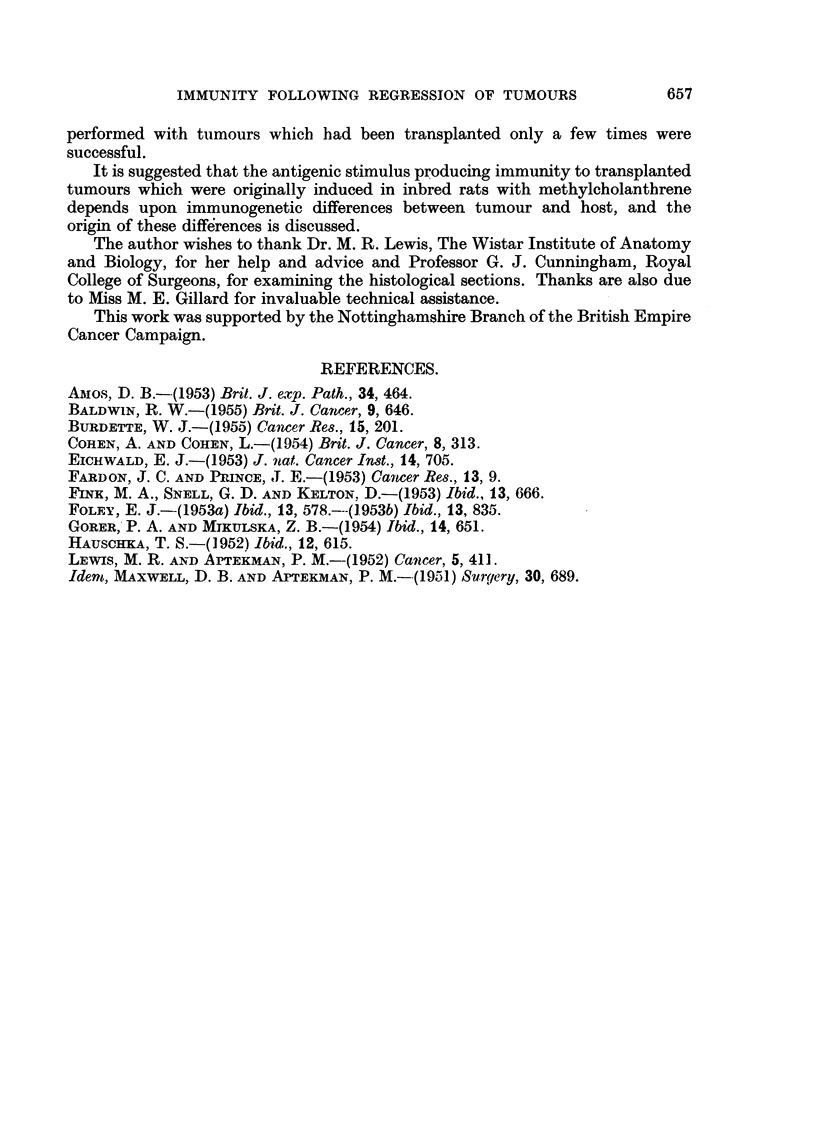# Immunity to Methylcholanthrene-Induced Tumours in Inbred Rats Following Atrophy and Regression of the Implanted Tumours

**DOI:** 10.1038/bjc.1955.70

**Published:** 1955-12

**Authors:** R. W. Baldwin


### IMMUNITY TO METHYLCHOLANTHRENE-INDUCED TUMOURS IN INBRED RATS FOLLOWING ATROPHY AND REGRESSION OF THE IMPLANTED TUMOURS.

R. W. BALDWIN.

*From The Cancer Research Laboratory, The University, Nottingham.*

Received for publication September 8, 1955.

IN a series of investigations using tumours which were originally induced with methylcholanthrene in inbred rats, it was shown that atrophy and regression of tumours caused by restricting their blood supply rendered the cured rats immune to subsequent inoculations of tumour, (Lewis, Maxwell and Aptekman, 1951 ; Lewis and Aptekman, 1952). Attempts to confirm these experiments using tumours which arose spontaneously in inbred strains of mice and had been subpassaged only a few times in animals of their strain of origin were unsuccessful (Fardon and Prince, 1953 ; Foley, 1953*a*) and it was suggested that the success of the earlier studies of Lewis and Aptekman (1952) was due to the presence of immunogenetic differences between tumour and host which probably arose during repeated transplantation of tumour. Later, however, Foley (1953*b*) showed that immunity could be induced in $C_3H$ mice following regression of tumours originally induced with methylcholanthrene, and so it would seem that the immune response evoked following tumour atrophy varies with the two types of tumour.

The development of tumour immunity in inbred rats following atrophy and regression of tumours originally induced with methylcholanthrene has been re-investigated and in the following experiments, a number of tumours have been studied at different stages of their transplantation history in an effort to determine the nature of the antigenic stimulus producing this immunity.

### MATERIALS AND METHODS.

The tumours described in a previous paper (Baldwin, 1955), together with three additional tumours, were used in the present experiments. These tumours were induced with methylcholanthrene in rats of selected inbred litters and each tumour was transplanted only into the offspring produced by mating litter mates of the tumour donor rat. The new tumours were designated as follows :

Sarcoma S67 : A very hard tumour which reached palpable size within 12 days of implantation in Group II rats and then grew very slowly until host rats had to be killed within 6 to 10 weeks.

Sarcoma S606 (Group 13 rats) and Sarcoma S609 (Group 12 rats) : Both tumours were moderately active, growing to palpable size within 8 days of implantation. Rats bearing these tumours became moribund within 4 to 6 weeks and were killed.

### EXPERIMENTAL METHODS.

The technique of tumour transplantation has been fully described in a previous paper (Baldwin, 1955). Rats bearing tumour grafts consisting of approximately one half trocar full of tumour tissue were examined three times a week; tumour sizes being measured in three dimensions. When engrafted tumours reached a size of approximately 20 × 10 × 10 mm. (Table I), they were ligated so as to restrict their blood supply. This was achieved in anaesthetised rats by firmly drawing a loop of suture nylon under the base of the tumour which was lifted free from the muscles on the back of the rat so that the tumour was enclosed within a fold of loose skin. Further loops of nylon were placed around the skin pockets at 24 hour intervals until the tumours regressed. Tumours usually became discoloured and considerably swollen within 24 hours of ligation. Then, following absorption of the tumours, the skin pockets slowly dried up over several days, leaving small scars at the tumour implant sites.

Rats in which ligated tumours had regressed were challenged for immunity 3 to 4 weeks later by re-implantation of the appropriate tumour. Challenge grafts

TABLE I.—*Inhibition of Tumour Growth following Ligation of Transplanted Tumours in Inbred Rats.*

| Tumour and litter of origin. | Transplant generation treated. | Number of days tumour had grown before treatment. | Number of tumour-bearing rats treated. | Number of tumours regressing. |
|---|---|---|---|---|
| S5 (Group B) | 19 | 6 | 5 | 5 |
| | 20 | 5 | 19 | 19 |
| | 22 | 4 | 6 | 6 |
| | | | 30 | 30 |
| S69 (Group I) | 9 | 5 | 10 | 5 |
| | 16 | 3 | 9 | 6 |
| | 19 | 4 | 11 | 11 |
| | 25 | 4 | 11 | 6 |
| | 26 | 4 | 7 | 6 |
| | | | 48 | 34 |
| S67 (Group II) | 4 | 17 | 3 | 3 |
| | 5 | 10 | 9 | 9 |
| | 6 | 10–14 | 17 | 17 |
| | 7 | 8 | 12 | 12 |
| | 8 | 10 | 9 | 9 |
| | | | 50 | 50 |
| S66 (Group III) | 8 | 5 | 3 | 2 |
| | 9 | 10 | 14 | 14 |
| | 10 | 7–10 | 15 | 12 |
| | 12 | 7 | 11 | 10 |
| | | | 43 | 38 |
| S609 (Group 12) | 2 | 6 | 8 | 8 |
| | 3 | 8 | 11 | 11 |
| | 4 | 7–10 | 16 | 15 |
| | 6 | 5 | 4 | 4 |
| | | | 39 | 38 |
| S606 (Group 13) | 2 | 6–8 | 13 | 12 |
| Total | | | 223 | 202 |

were made in the left side of the rat and were approximately half the size of the initial implant. Any rats proving to be resistant to the challenge grafts were re-implanted with large amounts of tumour (1 to 2 trocars full) 1 to 2 months later and kept under observation for at least 6 months. During this time the tumour implantation sites were examined twice weekly, and at the conclusion of each experiment a number of rats were examined for metastatic growth.

### RESULTS.

The results of experiments in which transplanted tumours in inbred rats were ligated in skin pockets so as to occlude their blood supply are recorded in Table I. In most cases it was possible to isolate completely the small progressively growing tumour grafts and so obstruct their blood supply. Occasionally, however, tumour grafts invaded the muscle tissue of the host so that it was not possible to ligate the whole tumour. In these experiments the tumours continued to grow even though the treatment destroyed practically all of the original tumour grafts. This often happened in experiments with the rapidly growing tumour S69 and also occasionally with tumour S66 (Table I).

The results recorded in Table II show that out of 197 rats in which tumours had regressed as a result of ligation only 77 (39 per cent) were resistant to the first challenge graft of the original tumour. However, the immune response evoked following atrophy and regression of the different tumours varied considerably and appeared to be related to the growth rate of the tumours. Thus regresssion of the rapidly growing tumour S69 induced tumour resistance in only 1 cured rat (3 per cent) and regression of two other fast-growing tumours, S5 and S606, produced similar results. In contrast, experiments performed with tumour S67, which had a relatively slow growth rate, were nearly always successful, immunity being produced in 76 per cent of the cured rats. The results obtained with tumours S66 and S609 were more variable, although approximately one half of the cured rats were found to be resistant to tumour growth.

As shown in Table II, repeated transplantation of tumour had no great influence on the ability of atrophying tumour to induce a resistant state. Thus in experiments with tumour S67, it was found that immunity could be induced using tumour grafts from the 4th to the 8th generation of transfer. Similar results were obtained in experiments where regression of tumour induced little or no tumour resistance. For example, regression of grafts of tumour S69 from between the 9th and 26th generation induced a resistant state in only one rat.

Most of the rats in which the first challenge graft had failed to grow were found to be immune to re-implantation with large doses of the appropriate tumour, and out of 77 rats challenged only 3 (4 per cent) proved to be susceptible to tumour growth.

### DISCUSSION.

The results obtained in the above experiments confirm the findings of Lewis and Aptekman (1952) and Foley (1953*b*) regarding the development of immunity in inbred animals to transplanted tumours originally induced with methylcholanthrene. However, the immune response evoked following atrophy and regression of different tumours was found to vary considerably, depending upon their growth rate. Thus regression of rapidly-growing tumours as a result of ligation induced

TABLE II.—*Development of Tumour Immunity in Inbred Rats following Induced Regression of Tumours Originating in the Same Strain.*

| Tumour and litter of origin. | Transplant generation used for first graft. | Number of rats in which tumours had regressed. | Number of cured rats immune upon re-implantation. | Per cent immune. | Transplant generation of tumour used for challenge graft. | Number of untreated rats inoculated.* |
|---|---|---|---|---|---|---|
| S5 | 19 | 5 | 3 | | 19 | 3 |
| (Group B) | 20 | 19 | 2 | | 21 | 8 |
| | 22 | 6 | 0 | | 23 | 8 |
| | | 30 | 5 | 17 | | 19 |
| S69 | 9 | 5 | 0 | | 14 | 10 |
| (Group I) | 16 | 6 | 0 | | 17 | 4 |
| | 19 | 9 | 1 | | 21 | 6 |
| | 25 | 6 | 0 | | 26 | 10 |
| | 26 | 6 | 0 | | 27 | 5 |
| | | 32 | 1 | 3 | | 35 |
| S67 | 4 | 3 | 3 | | 5 | 9 |
| (Group II) | 5 | 9 | 9 | | 5 | 4 |
| | 6 | 16 | 15 | | 6 | 4 |
| | 7 | 12 | 5 | | 7 | 5 |
| | 8 | 9 | 5 | | 8 | 3 |
| | | 49 | 37 | 76 | | 25 |
| S66 | 8 | 2 | 1 | | 8 | 5 |
| (Group III) | 9 | 14 | 8 | | 9 | 9 |
| | 10 | 12 | 0 | | 10 | 4 |
| | 12 | 8 | 8 | | | 10 |
| | | 36 | 17 | 47 | | 28 |
| S609 | 2 | 8 | 8 | | 2 | 4 |
| (Group 12) | 3 | 11 | 6 | | 3 | 9 |
| | 4 | 16 | 1 | | 4 | 3 |
| | 6 | 3 | 2 | | 7 | 3 |
| | | 38 | 17 | 45 | | 19 |
| S606 (Group 13) | 2 | 12 | 0 | 0 | 2 | 2 |
| Total | | 197 | 77 | 39 | | 128 |

* Tumour grew in all untreated animals.

little or no tumour immunity, whereas treatment of tumour S67, which has a slow growth rate, rendered nearly all the cured hosts resistant to further implants of the original tumour. It should be pointed out that the method of testing for tumour immunity by re-implantation of tumour is an all-or-none technique which will not detect slight levels of resistance. In addition, the minimum level of resistance which will protect a cured host against re-implants of tumour will depend in part upon the growth rate or " virulence " of the tumour, and so this may explain why experiments with rapidly-growing tumours were unsuccessful.

The nature of the antigenic stimulus evoking an immune response following regression of transplanted tumour in inbred animals is still unknown, although it is now generally assumed that immunogenetic differences between tumour and host rather than specific tumour antigens are responsible for the induction of tumour immunity (Hauschka, 1952 ; Eichwald, 1953 ; Cohen and Cohen, 1954). Evidence to support this assumption has been obtained by Foley (1953*a*, 1953*b*), who showed that immunity could be induced in inbred mice against transplanted tumours which were induced originally with methylcholanthrene, but not against tumours which originated spontaneously in the same strain of mice.

According to Hauschka (1952) these immunogenetic differences arise during repeated subpassaging of tumour over long periods of time by mutation of either the tumour or host strain of animals. Although heterogeneity in the host strain of animals cannot be ruled out as a contributory factor, Foley (1953*a*) showed that this factor did not influence the immune response induced in mice following regression of transplanted tumours which arose spontaneously, and no immunity was produced. Repeated subpassaging of tumour also appears to have no great influence on the ability of atrophying tumour to induce tumour resistance, and in the present experiments, as well as those of Foley (1953*b*), it was possible to induce immunity before repeated tumour transplants had been made. Similarly, in the present study, attempts to induce immunity against a rapidly-growing tumour, S69, were nearly always unsuccessful even though experiments were performed with tumour grafts from between the 9th and 26th generation of transfer.

Thus if the induction of tumour immunity depends upon immunogenetic differences between tumour and host, then it would seem that the original induced tumour represents a mutated tissue capable of eliciting an immune response when implanted into the host strain of animals. This suggestion will require further study however, especially in view of Burdette's recent criticism of the correlation between mutagenicity and carcinogenicity of chemical compounds (Burdette, 1955).

A number of techniques have been described recently by which the antibody response to tumour inoculation can be determined. These include agglutination reactions using erythrocytes (Gorer and Mikulska, 1954) and leucocytes (Amos, 1953) and the anaphylaxis technique which was used by Fink, Snell and Kelton (1953) to determine the antibody response of Balb. C mice following inoculation of an homologous tumour. It may well be that the application of these techniques to the study of the antigenic structure of transplanted tumours which originated spontaneously or were induced with methylcholanthrene in inbred animals will disclose the nature of the antigenic stimulus producing immunity following induced regression of the latter type of tumour.

### SUMMARY.

Immunity to transplanted tumours originally induced with methylcholanthrene has been produced in inbred rats following atrophy and regression of tumour grafts ligated so as to restrict their blood supply.

The immune response evoked following regression of tumour was found to vary considerably with different tumours. In some cases regression of tumour induced immunity in practically all cured rats, whereas with other tumours none of the cured rats were resistant to re-implants of the original tumour. Repeated subpassaging of tumour did not influence the ability of atrophying tumour to induce tumour resistance, and in cases where it was possible to induce immunity experiments performed with tumours which had been transplanted only a few times were successful.

It is suggested that the antigenic stimulus producing immunity to transplanted tumours which were originally induced in inbred rats with methylcholanthrene depends upon immunogenetic differences between tumour and host, and the origin of these differences is discussed.

The author wishes to thank Dr. M. R. Lewis, The Wistar Institute of Anatomy and Biology, for her help and advice and Professor G. J. Cunningham, Royal College of Surgeons, for examining the histological sections. Thanks are also due to Miss M. E. Gillard for invaluable technical assistance.

This work was supported by the Nottinghamshire Branch of the British Empire Cancer Campaign.

### REFERENCES.

Amos, D. B.—(1953) *Brit. J. exp. Path.*, **34**, 464.
Baldwin, R. W.—(1955) *Brit. J. Cancer*, **9**, 646.
Burdette, W. J.—(1955) *Cancer Res.*, **15**, 201.
Cohen, A. and Cohen, L.—(1954) *Brit. J. Cancer*, **8**, 313.
Eichwald, E. J.—(1953) *J. nat. Cancer Inst.*, **14**, 705.
Fardon, J. C. and Prince, J. E.—(1953) *Cancer Res.*, **13**, 9.
Fink, M. A., Snell, G. D. and Kelton, D.—(1953) *Ibid.*, **13**, 666.
Foley, E. J.—(1953*a*) *Ibid.*, **13**, 578.—(1953*b*) *Ibid.*, **13**, 835.
Gorer, P. A. and Mikulska, Z. B.—(1954) *Ibid.*, **14**, 651.
Hauschka, T. S.—(1952) *Ibid.*, **12**, 615.
Lewis, M. R. and Aptekman, P. M.—(1952) *Cancer*, **5**, 411.
*Idem*, Maxwell, D. B. and Aptekman, P. M.—(1951) *Surgery*, **30**, 689.